# Driving Assistance System with Obstacle Avoidance for Electric Wheelchairs

**DOI:** 10.3390/s24144644

**Published:** 2024-07-17

**Authors:** Esranur Erturk, Soonkyum Kim, Dongyoung Lee

**Affiliations:** 1 Department of Computer Science, Faculty of Engineering, Lund University, Box 118, 221 00 Lund, Sweden; esranur.erturk@cs.lth.se; 2 Center for Intelligent and Interactive Robotics, Korea Institute of Science and Technology (KIST), Seoul 02792, Republic of Korea; kim.soonkyum@kist.re.kr

**Keywords:** smart electric wheelchair, LiDAR sensor, dynamic window approach, obstacle avoidance, driving assistant system

## Abstract

A system has been developed to convert manual wheelchairs into electric wheelchairs, providing assistance to users through the implemented algorithm, which ensures safe driving and obstacle avoidance. While manual wheelchairs are typically controlled indoors based on user preferences, they do not guarantee safe driving in areas outside the user’s field of vision. The proposed model utilizes the dynamic window approach specifically designed for wheelchair use, allowing for obstacle avoidance. This method evaluates potential movements within a defined velocity space to calculate the optimal path, providing seamless and safe driving assistance in real time. This innovative approach enhances user assistance and safety by integrating state-of-the-art algorithms developed using the dynamic window approach alongside advanced sensor technology. With the assistance of LiDAR sensors, the system perceives the wheelchair’s surroundings, generating real-time speed values within the algorithm framework to ensure secure driving. The model’s ability to adapt to indoor environments and its robust performance in real-world scenarios underscore its potential for widespread application. This study has undergone various tests, conclusively proving that the system aids users in avoidance obstacles and ensures safe driving. These tests demonstrate significant improvements in maneuverability and user safety, highlighting a noteworthy advancement in assistive technology for individuals with limited mobility.

## 1. Introduction

In recent times, the importance of wheelchairs has been increasing for individuals facing mobility issues, allowing them to perform daily activities and sustain their lives independently. Various types and designs of electric wheelchairs have been produced to meet the needs of users [1,2]. The use of some developed electric wheelchairs can be challenging and may necessitate additional training, such as teaching users how to operate all functions of the wheelchairs. Moreover, certain multifunctional electric wheelchairs can be relatively expensive, making them less accessible to individuals with mobility challenges. Some electric wheelchairs may also hinder the user’s movement due to the addition of sensors and components that can interfere with wheelchair design [3,4]. This could create discomfort for the user. Additionally, in some cases where users experience physical difficulties and lack sufficient strength, manual wheelchairs may not fully meet their needs. To address such situations and provide users with a more comfortable life, user-friendly electric wheelchairs that can be easily obtained have been developed.

Electric wheelchairs significantly enhance the quality of life for users experiencing mobility issues. Additionally, many wheelchairs on the market today are customized with various features to better meet the users’ needs. Thus, electric wheelchairs can be made more functional according to users’ requirements and before electric wheelchairs are introduced to users, they undergo various projects and research to achieve specific goals [1,3,5,6,7,8]. One of these research areas involves the development of an autonomous and real-time operating electric wheelchair, and the goal is to navigate dynamic obstacles while avoiding collisions to reach the destination point [8,9]. This study employs LiDAR sensors and artificial potential fields (APFs). However, a disadvantage of this study is the tendency of the electric wheelchair to get stuck in local minimum points as it approaches the destination. Therefore, when considering the use of this algorithm in an autonomous electric wheelchair, it may lead to the wheelchair getting stuck or entering loops in certain areas.

Rui Zhang et al. [10] present a study on BCI (brain–computer interface)-controlled wheelchairs. Instead of creating a system entirely controlled by BCI, this study employs an automatic path-planning system. After scanning the environment, the wheelchair prompts the user for the destination entered via BCI. The wheelchair then navigates toward the predetermined destination, and the user can stop the wheelchair at any time using the BCI interface.

Another example of a study involves the development of a motor imagery (MI) brain–computer interface (BCI) application and testing wheelchair control [11]. This study demonstrates acceptable performance in navigation. However, one of its weaknesses lies in the low accuracy rates of MI decoders. The low accuracy rates may lead users to struggle with issuing desired commands while controlling the wheelchair, consequently impacting user experience negatively. Furthermore, this study suggests a control schema for users, necessitating users to learn this control schema.

Research [12] encompasses a voice-controlled electric wheelchair design and control based on speech recognition algorithms. An adaptive neuro-fuzzy controller is employed to generate real-time control signals for wheelchair movement. The accuracy of the speech recognition algorithms in this study may be debatable as misinterpreted commands could affect user experience. Additionally, network connections within the system in this study may prevent users from establishing communication in emergency situations.

Wheelchair users facing mobility issues often encounter a fundamental challenge related to the control mechanism [5,13,14,15,16]. Moreover, automation plays a significant role in wheelchairs in many studies. The literature also aimed to use more complex control systems such as BCI directly involving the user’s body instead of a joystick [10,17,18]. However, numerous studies have complicated wheelchair usage from a technical and hardware perspective. These control systems and approaches are often developed based on the user’s specific disability, focusing on overcoming a particular obstacle, and while many of these studies have proven to offer excellent solutions for individual users, there is a scarcity of systems that transform conventional electric wheelchairs into a new form without causing discomfort to all users, without creating movement obstacles, and without requiring an additional control mechanism. Users either need to acquire a new wheelchair or learn to use a more complex control mechanism.

The designed mechanism is intended for indoor use and can be easily attached and detached. Typically, initiatives for indoor applications are constrained. Therefore, with this mechanism, existing manual wheelchairs can be converted into electric wheelchairs without the need to purchase a new one. Additionally, with the developed algorithm that controls the system and provides obstacle avoidance, the indoor electric wheelchair is transformed into a smart electric wheelchair. With this algorithm, the user can easily move the wheelchair using a joystick. Furthermore, the sensors located on the front, right, and left sides of the wheelchair serve to protect the user from various collisions. This system is fundamentally a user-assistance driving system that prevents potential collisions during user driving. Moreover, since this system is designed with user-centric principles, users do not require additional training to use it. Only the necessary system is attached to the existing manual wheelchair, and the user can continue to use it in their daily life.

The primary goal is to ensure the safety of the system and facilitate real-time movement by identifying obstacles in the path of motion and generating appropriate paths to navigate around them [19,20]. To achieve this goal, various algorithms and approaches have been developed [18,21,22,23,24,25]. The most popular algorithms commonly used for obstacle avoidance in mobile robots include the dynamic window approach (DWA), vector field histogram (VFH), and artificial potential fields(APF) [26].

The VFH is commonly employed in obstacle avoidance and path planning algorithms [27]. The VFH algorithm involves dividing data collected from integrated sensors or laser scanners on the robot into angular sectors. It calculates the number of data points or measurements within specific angular ranges for each sector. Using these measurements, a histogram known as the vector field histogram is constructed, representing the distribution of the robot’s surroundings, with each segment corresponding to an angular sector [27,28]. The VFH algorithm analyzes this histogram to generate a path, allowing the robot to navigate its environment by considering the proximity and positions of obstacles.

One feature that sets VFH apart from DWA is its more complex structure, relying on sensory data and the analysis of a generated histogram [28]. Therefore, in terms of computation, DWA is considered a more efficient approach. Since VFH heavily depends on predefined angular sectors and histogram bins, variations in parameters can significantly impact the algorithm’s performance. However, DWA is a more adaptable approach to systems as it dynamically responds to current conditions and the robot’s constraints.

Another widely used approach for obstacle avoidance and path planning is the APF. The APF algorithm operates by simulating forces exerted on the robot to influence its movement. It characterizes the nearby environment with attractive and repulsive forces, aiming to guide the robot toward the target location and avoid obstacles [29]. The attractive force draws the robot toward the goal, while the repulsive forces push it away from obstacles [29]. The combination of these forces creates a virtual field, allowing the robot’s movement.

One significant advantage of APF, similar to DWA, is its real-time operation capability. However, due to the gradient descent structure of APF, the algorithm may become trapped in suboptimal paths and local minima [28,30,31]. If the balance between attractive and repulsive forces is not properly calibrated during algorithm usage, the robot may encounter difficulties in navigation in narrow and complex areas. Additionally, APF typically focuses on position control rather than velocity control. Since velocity control is an important aspect of this study, the use of DWA is a better option.

The DWA is a technique designed to generate optimal motion plans, considering both the mobility capabilities of the robot and the constraints imposed by the environment [21,22,26,32,33]. This method employs a dynamic window that is adjusted based on the robot’s dynamics and environmental limitations, allowing the calculation of possible movements within a specific time interval. The dynamic window is continuously updated depending on the current speed and position of the robot, providing real-time outputs and enabling the robot to perform obstacle avoidance effectively. In summary, DWA is commonly used in robotic systems, offering real-time action planning [22,26].

When compared to other algorithms, the decision to use DWA for controlling and providing obstacle avoidance in the module of this study has been deemed appropriate. DWA aims to find the optimal path by sampling various velocity pairs in velocity space while avoiding obstacles. This approach calculates speed values based on user commands received through the joystick and creates a safe path by detecting obstacles. The efficiency and simplicity of DWA in real-time applications are significant advantages.

However, the literature indicates that metaheuristic-based neural networks (e.g., RNN-based metaheuristic approaches) are used to optimize the cost functions of nonlinear control systems. These methods perform particularly well in complex and dynamic environments and achieve high accuracy when trained with large datasets. Despite their high performance, their use in real-time applications is challenging due to computational costs and implementation difficulties, which can delay the electric wheelchair’s response to instantaneous speed changes. These differences between DWA and metaheuristic-based neural networks clarify why DWA was the preferred method in our project and demonstrate how this method can be effectively used in electric wheelchair systems.

In conclusion, the installation of the sensors and the driving module used in this study can be easily accomplished, and this setup can be applied to multiple wheelchairs. Furthermore, by enhancing DWA to provide obstacle avoidance, a new algorithm has been designed to create an assistance system for the user. The algorithm’s performance has been verified through testing.

## 2. System and Structure

### 2.1. Electric Wheelchairs

**Figure 1:**
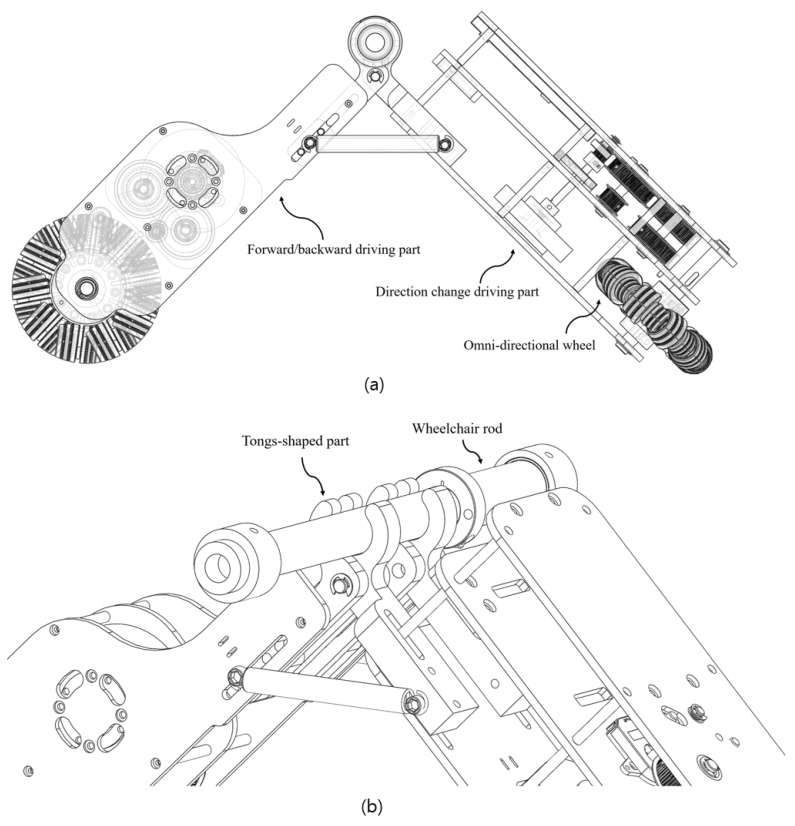
Detachable driving module for electrification of manual wheelchairs: (**a**) overall driving module, (**b**) tongs-shaped part.

A new driving module has been designed to control the electric wheelchair. The driving module was designed by dividing it into a forward and backward driving unit and a direction change driving unit as shown in Figure 1a. At this time, the wheels were applied with omnidirectional wheels so that there was no interference with each other. When the wheelchair is placed on the driving module as shown in Figure 1b for the mounting of the driving module, the tongs-shaped part of the module holds the rod of the wheelchair and firmly engages the module. At this time, if the module is not mounted in the center of the rod of the wheelchair, driving is affected. At this time, the external force acting on the wheelchair is caused. In other words, according to the mounting position of the driving module, an external force that rotates the wheelchair is caused. Therefore, driving evaluation was performed according to the position of the module developed in the previous study, and a control algorithm to overcome this was proposed. It was verified through experiments and showed the usefulness of the algorithm through statistical analysis. In addition, verification was completed through an external certification body for the performance evaluation of the module [1].

**Figure 2:**
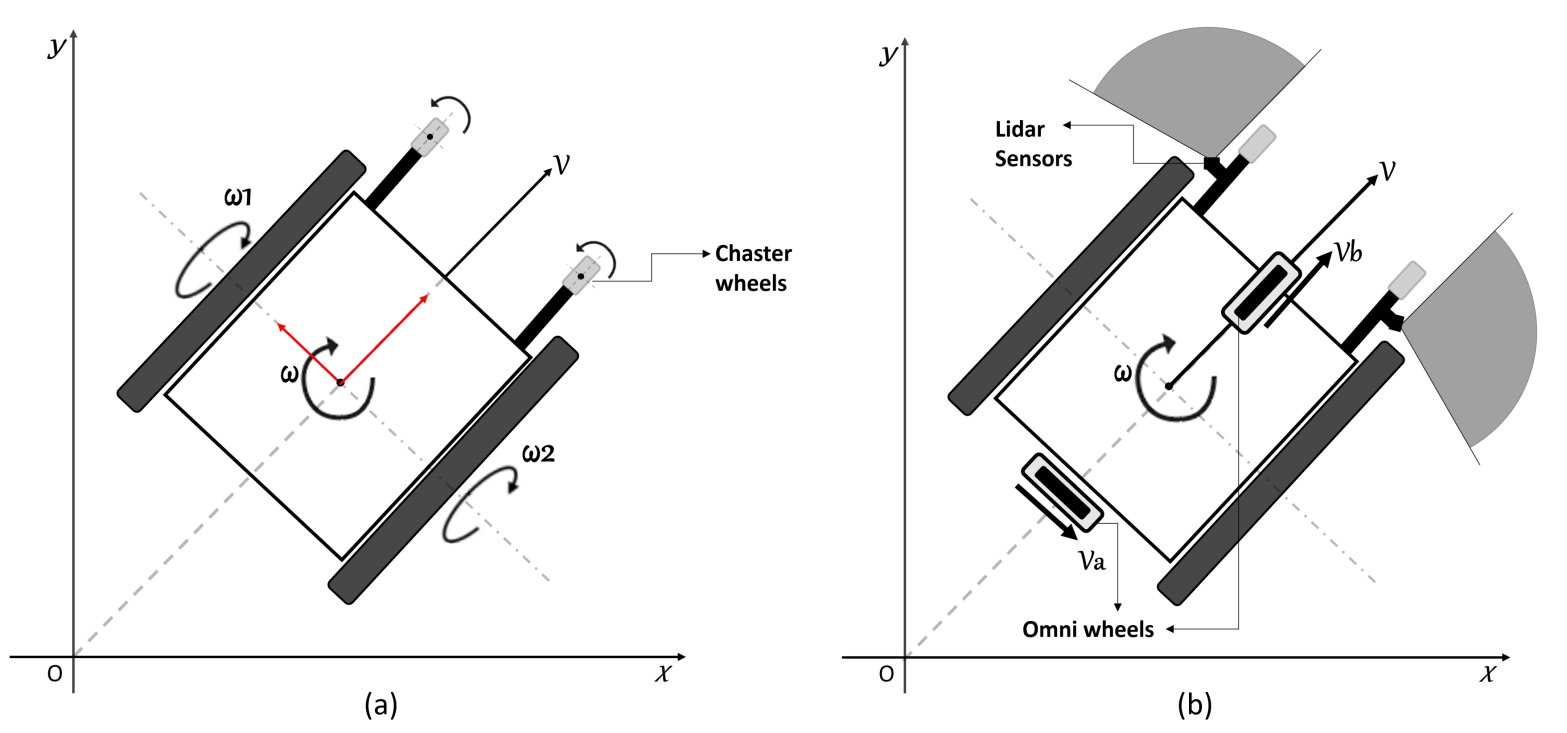
(**a**) Common 2-wheel differential drive model (**b**), electric wheelchair model designed for use in this study.

**Figure 3:**
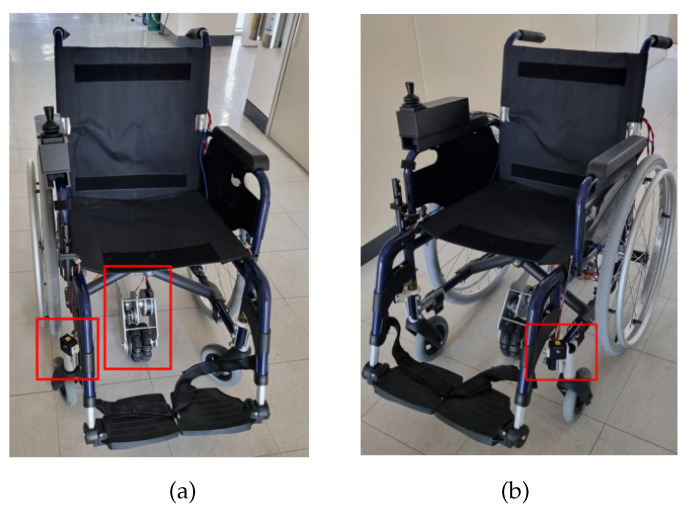
Overall wheelchair system equipped with driving module: (**a**) front view, (**b**) side view.

Figure 2 illustrates the coordinate system used in traditional electric wheelchairs, which commonly employ a differential driving approach for versatile maneuvering, allowing both rotational and linear movements [1]. In this configuration, the wheelchair’s forward/backward motion is determined by the direction, while the angular velocities (ω1 and ω2) necessary for turning are influenced by the speed difference between the two wheels. This approach is commonly employed in the design of most electric wheelchairs [1]. As a result, in this study, a new driving model has been designed for the control of the smart electric wheelchair, and an algorithm based on DWA has been developed to prevent collision occurrences during user driving. The utilized electric wheelchair is shown in Figure 3.

#### 2.1.1. System Model

In this study, a flat-type brushless direct current (BLDC) motor without a Hall sensor was chosen due to the size of the driving module [1]. Figure 2a illustrates a general model of a two-wheeled wheelchair [24]. Current designs of electric wheelchairs generally incorporate the use of omnidirectional or mecanum wheels, and the configuration of these wheels and the operation of each wheel are critical to facilitate changes in motion and direction. Standard manual wheelchairs employ a differential drive method to achieve both angular and linear motion. Consequently, by utilizing the speed differential between the wheels, wheelchair movement is directed using the forward/backward propulsion speed and the rate of change in orientation. In other words, when a person rides a manual wheelchair and changes direction, they use the difference in speed between the two wheels or rotate the two wheels in different directions to change the direction of the wheelchair. However, in the case of the existing electric wheelchair, the radius of rotation changes because only the difference in speed between the two wheels is used. However, our driving module has a different shape, but the same principle as the differential drive model of the existing manual wheelchair (Figure 2a) is applied. That is why we developed a driving module of a differential drive model for the same behavior as a manual wheelchair.

The driving model shown in Figure 2b was implemented in the electric wheelchair used in this study. This driving model incorporates two omni wheels, where one enables backward and forward movement, and the other supports directional changes, facilitating angular motion [1,34]. The driving module provides forward and backward movement (*V*b) as well as changes in direction (*V*a), and these functions operate independently of each other. The movement of the wheelchair is achieved through the separate control of the two omnidirectional wheels via joystick assistance.

In summary, a new driving module (Figure 2b) has been developed, and a study has been conducted using this module’s control to achieve obstacle avoidance. This obstacle avoidance process has been achieved by modifying and adapting the commonly used DWA in mobile robots to function in this context. The newly developed algorithm demonstrated, during test drives, that it operates synchronously with the new driver module, making real-time speed adjustments when objects are detected.

The electric wheelchair used in this project is designed to assist the user, and while providing assistance to the user, the electric wheelchair aims to detect and prevent various collisions and obstacles that may arise in situations where the user cannot see or predict. In this study, the DWA has been employed for collision avoidance. Although this model is commonly used for mobile robots, a similar algorithm has been established, considering the general structure of DWA, and a system has been designed. This is because the motion of the electric wheelchair is controlled by the user’s requests through a joystick, representing a driving assistance system rather than a fully autonomous system.

The operation of the electric wheelchair fundamentally involves the user controlling it using a joystick, and this information is transmitted to the Raspberry Pi, where the algorithm processes the data resulting in the generated motion. When the user manipulates the joystick, the Raspberry Pi receives the axis information required for the desired direction and simultaneously analyzes the surroundings using data from LiDAR sensors [34]. After processing these inputs in the controller, the output of the algorithm determines linear and angular velocity values to ensure safe navigation. These values are then communicated to the motor driver via serial communication to drive the motion of the omnidirectional wheels.

**Figure 4:**
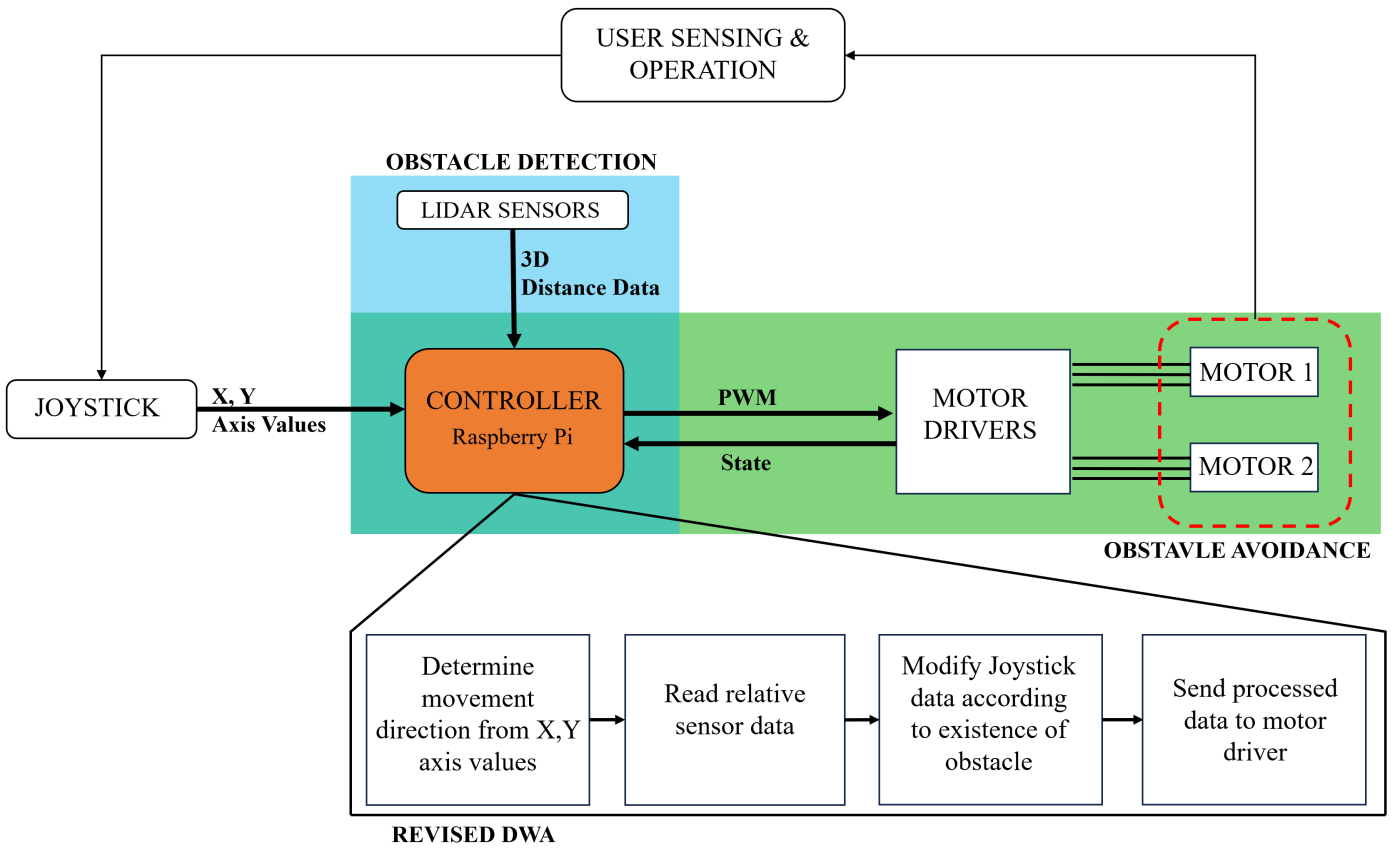
Model framework of smart electric wheelchair system.

Figure 4 illustrates the model framework of this study, revealing the two fundamental steps: obstacle avoidance and object detection. As depicted in the figure, while the user controls the wheelchair using axis information from the joystick, simultaneously, 2D distance data resulting from the LiDAR sensor’s environmental scan are transmitted to the Raspberry Pi. In this study, apart from a system assisting the user, a low-cost system is intended; therefore, Raspberry Pi is utilized due to its capacity to control all tasks at a low cost. The information from the sensor and joystick constitutes the inputs for the developed DWA algorithm running on the controller. This algorithm, based on the current data, generates linear and angular velocity values. The algorithm uses data from sensors to determine speed values, enabling obstacle avoidance and preventing potential collisions in the presence of obstacles. To convey information to the motor driver, the Raspberry Pi employs serial communication for interaction. The speed details transmitted through this communication channel govern the motion of the driving module, which incorporates omni wheels.

#### 2.1.2. Sensors

In this study, considering a cost-effective system, a 2D/3D Dual CygLiDAR sensor was used to perceive environmental information. The sensor is strategically placed on the front right and left sides of the electric wheelchair to avoid hindering the user’s movement. The key features of the utilized sensor are outlined in Table 1.

The LiDAR sensors can perform scans concurrently in both 2D and 3D. However, for this study, it was decided to utilize 2D distance measurement, considering that longer-range measurements would be more accurate. This decision allows the sensor to detect environmental data for longer-range objects.

**Figure 5:**
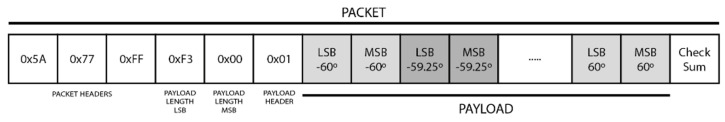
Data packet structure for 2D scanning.

The scanning process begins by sending a request packet to the sensor, instructing it to perform a 2D scan and transmit the resulting data. Subsequent to this request, the response packet from the sensor is examined, consisting of a 329-byte array containing header information, sensor readings, and a checksum. The structure of the data packet transmitted by the sensor for 2D scanning is depicted in Figure 5. In this operational mode, the sensor conducts a scan covering 120 degrees at a precision of 0.75 degrees [35]. Each angle is encoded by 2 bytes—specifically, the Most Significant Byte (MSB) and the Least Significant Byte (LSB). With a resolution of 0.75 degrees, the data encompass 322 samples, ranging from −60 degrees to +60 degrees (inclusive of 0), detailed in the payload segment of the packet.

To process the acquired data, each pair of MSB and LSB bytes is combined to calculate the distance value in millimeters corresponding to each angle. These angle–distance pairs are concatenated into a data string for subsequent analysis. This array of distance data represents the 120-degree scan. Upon examination of these data, if the values fall below a predefined threshold (set at 70 cm in this investigation), it is interpreted as indicative of an obstacle at that specific point.

The positions and angles of the electric wheelchair’s sensors, along with the current angle under analysis, are used to determine the coordinates of the detected obstacle relative to the wheelchair’s center. These identified coordinates are gathered and organized into an array, forming a point cloud representation. This point cloud, created from the collected obstacle information, is then utilized in the developed DWA algorithm.

This method simplifies the processing of sensor data and reduces computational costs while providing sufficient accuracy for safe path planning. Additionally, since a Raspberry Pi was used in the project, employing 2D distance measurements allows for faster processing and quicker algorithm responses. Therefore, 2D measurements were found to be more suitable and practical than a more complex approach.

### 2.2. Dynamic Window Approach

The DWA is a technique designed to facilitate the generation of optimal motion plans, taking into account both the mobility capabilities of the robot and environmental constraints [22,26,36,37,38,39]. With this approach, a window is created representing the robot’s current state within the allowed speed intervals. By adjusting this window based on dynamic and environmental constraints, the possible movements the robot can make within a specific time frame are defined [40]. This window is continuously updated based on the robot’s current speed and position. Consequently, DWA is a frequently used approach in the implementation of real-time systems [22,26].

To utilize this algorithm, there are essentially two crucial steps. In the first step, a search space is created by considering the robot’s specified motion constraints and the speed range it can reach. This determines the comprehensive potential speeds the robot can achieve.

In the second step, the algorithm selects the speed pair within this search space that minimizes the cost function. This speed pair is considered optimal because the cost function assists the robot in determining the most suitable path within its current environmental factors, and the robot’s motion is optimized with these speed pairs.

## 3. Modification of DWA for Obstacle Avoidance

The fundamental goal of the DWA algorithm is to find the optimal path for mobile robotic systems, ensuring obstacle avoidance [22,26,38,39]. However, in this study, the designed driving module and algorithm aim to provide driving assistance to the user. Therefore, the existing inputs were considered, and the DWA was modified to be suitable for this project.

In the traditional DWA algorithm, the path of the mobile robot within each speed cluster is examined towards the conclusion of a specific duration. It assesses the velocity clusters within the velocity space and considers the mobile robot’s motion model. Using the formulated evaluation function, the algorithm identifies the most appropriate trajectory among these and selects the corresponding speed set. Consequently, the motion of the mobile robot is facilitated by devising path planning based on the speed pair aligned with the optimal trajectory.

In the developed DWA for this study, the algorithm has been adapted to the movements of the electric wheelchair. Instead of focusing on target points and trajectories, the algorithm concentrates on the changing speed values of the wheelchair. Based on the axis information from the joystick, the algorithm provides appropriate speed values as output.

### 3.1. Motion Model

The simple differential drive robot model is a suitable and practical approach for electric wheelchairs. The kinematic model of electric wheelchairs is often represented as a simple differential drive robot model. This model is commonly used to describe the movement of a two-wheeled robot. Electric wheelchairs also employ a differential drive system, where the two wheels can be independently controlled to provide steering. This is analogous to the operation of differential drive robots. Therefore, it is believed that this model sufficiently represents the fundamental motion dynamics of a wheelchair. Additionally, our algorithm developed with DWA is used for obstacle avoidance and real-time speed adjustments according to the surrounding conditions. Hence, this method was chosen because it is deemed to work effectively with electric wheelchairs.

The DWA algorithm samples linear and angular speeds within a specified window area, considering the hardware limitations and kinematic body constraints of the electric wheelchair [22,41]. Consequently, the first step involves constructing a kinematic model for the electric wheelchair, as illustrated in Equation (1). When determining the subsequent position for each velocity set, the starting point is considered (0, 0). Due to the absence of a global position, the algorithm computes the next position relative to its current location.

(1) $$\begin{array}{cc} x(t) & =x\left(t-1\right)+v\left(t\right)\cdot \cos\theta_{t-1}\cdot T\\ y(t) & =y\left(t-1\right)+v\left(t\right)\cdot \sin\theta_{t-1}\cdot T\\ \theta_{t} & =\theta_{t-1}+\omega \left(t\right)\cdot T \end{array}$$

Before calculating the distance cost in the algorithm, the anticipated position that the wheelchair will reach is computed with the current velocity values. This involves utilizing the specified prediction time and *T* values from the configuration (*T* = 0.1). The linear and angular speed values are appropriately processed to align with the duration *T*. This process entails calculating the linear position based on the y-coordinate using the linear speed and performing trigonometric calculations based on the x-position and angle using the angular speed. Through these equations, the velocity position of the electric wheelchair after time *T* is determined. This cumulative process persists until the total time matches the prediction time duration, and as a result, the obtained final position represents the endpoint reachable by the electric wheelchair with its current speed at that moment.

### 3.2. Distance Penalty Calculation

The concept of distance penalty is widely used in robotic systems to ensure obstacle avoidance. The logarithmic distance penalty function employed in this study can be described as an adaptation of existing methods tailored to the requirements of an electric wheelchair. The logarithmic function provides a safety mechanism for the user by rapidly increasing the penalty as the electric wheelchair approaches an obstacle. In summary, as mentioned above, the use of penalties to prevent collisions is a commonly used method in the literature, but it is a formula customized according to the specific project. In addition to the algorithm, another developed approach is the creation of a distance penalty. To find the penalty, firstly, the distance function needs to be calculated, and this function is determined by calculating the distance between the objects in the point cloud scanned with LiDAR and the estimated next position of the electric wheelchair.

**Figure 6:**
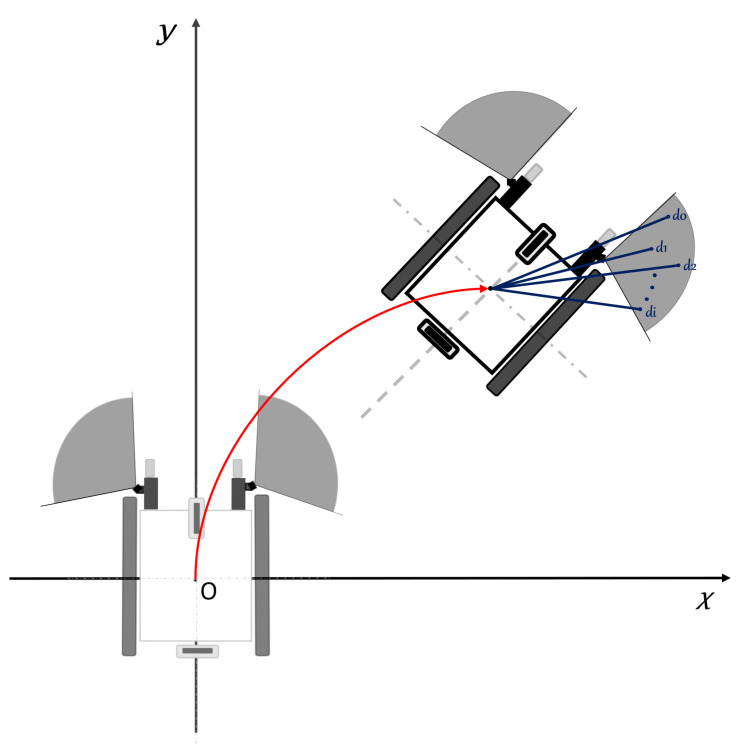
The recognition of obstacle by the electric wheelchair.

Figure 6 represents the point cloud of sample points created by scanning the environment with LiDAR sensors. The algorithm calculates the minimum distance to the next position of the electric wheelchair among these points, selecting the nearest obstacle, as shown in Equation (2).

(2) $$\mathrm{dist}=\min(d_{i})$$

Once the distance to the nearest object is calculated, this value is used to compute the distance penalty in equation below:

(3) $$d_{\mathrm{penalty}}=\max\left(-\log\left(\mathrm{dist}/D\right),0\right)$$

**Figure 7:**
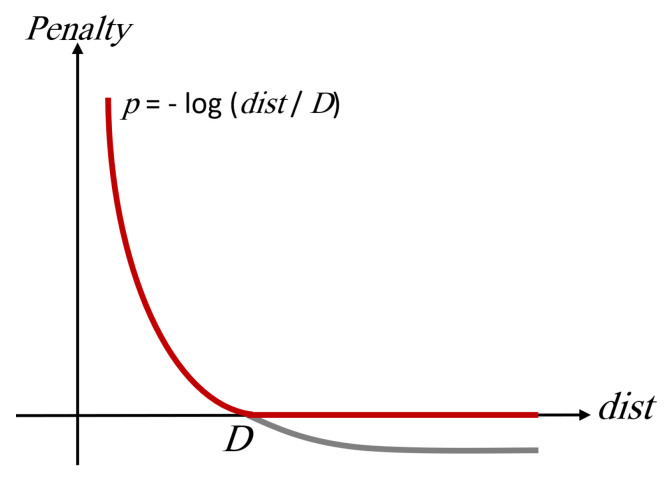
Distance penalty calculation and impact of D.

The *D* in the distance penalty equation is a design parameter used to adjust the sensitivity of the electric wheelchair to obstacles in its surroundings. The impact of the *D* value on the equation is shown in Figure 7. According to this graph, higher values of *D* approach zero, indicating no collision, and as *D* approaches zero, the distance penalty exponentially increases towards infinity. However, as long as *D* is not zero (*D* > 0), a collision will not occur. The variation of the *D* value can alter the sensitivity of the electric wheelchair to objects in its surroundings.

### 3.3. Velocity Space

Within the hardware constraints of the electric wheelchair, there are numerous speed sets. However, due to these hardware limitations, there exist maximum and minimum speed constraints [22,38,41].

(4) $$V_{all}=\left(v,\omega \right)|v\in \left[v_{\min},v_{\max}\right]⋂\omega \in \left[\omega_{\min},\omega_{\max}\right]$$

As shown in Equation (4), there are both minimum and maximum values for both linear and angular speeds, and the range encompassing these values is denoted as $V_{\mathrm{all}}$. In consideration of the physical dynamics of the motor and safety, for this study, the maximum and minimum speed values for linear speed are [−0.28, +0.28] *m/s*, and for angular speed, they are [−0.75, +0.75] *rad/s*. Additionally, to account for the accelerations applied by the motor, the achievable speeds of the wheelchair in the next time interval are constrained within a specific range, reducing it to a dynamic window, as represented by $V_{\mathrm{dw}}$ in the equation below:

(5) $$V_{\mathrm{dw}}=\left(v,\omega \right)|v\in \left[v_{0}-{\dot{v}}_{a}\Delta t,v_{0}+{\dot{v}}_{a}\Delta t\right]\wedge \omega \in \left[\omega_{0}-{\dot{\omega }}_{a}\Delta t,\omega_{0}+{\dot{\omega }}_{a}\Delta t\right]$$

In this equation, *t* represents the next time interval, $v_{a}$ and $\omega_{a}$ denote accelerations, $v_{0}$ and $\omega_{0}$ represent current velocities. As can be understood from this, the dynamic window is constrained by current velocities and the values these velocities will reach as a result of acceleration. Other speed values outside this dynamic window are not considered for evaluation since it is known that they cannot be reached in the next time interval, making them irrelevant for obstacle avoidance.

As a result, the dynamic window constraint reduces the speed space to a specific area, allowing calculations only on certain speeds for obstacle avoidance. Finally, the speed space is reduced, as shown in the following equation:

(6) $$S_{vs}=V_{all}\cap V_{dw}$$

### 3.4. Cost Function

After creating the search space for velocities $S_{\mathrm{vs}}$, a velocity set within this space is selected to minimize the cost function. To minimize the cost function, the velocity and distance penalty values are processed, and optimal linear and angular speed values are chosen. The calculation of the cost function is shown in the equation below:

(7) $$\mathrm{Cost}\left(v,w\right)=\alpha \;|v-v_{d}|+\beta |\omega -\omega_{d}|+\gamma \;d_{\mathrm{penalty}}\left(v,w\right)$$

In this function, alpha, beta, and gamma are weight coefficients and optimal weight values have been adjusted during the testing process to consider the sensitivity of the variable. $v_{d}$ and $w_{d}$ represent, respectively, the linear and angular speed values obtained from the joystick. The *x*-axis and *y*-axis values from the joystick are multiplied by the maximum speeds to calculate $v_{d}$ and $w_{d}$, as shown in the following equation:

(8) $$\begin{array}{cccc} v_{d} & =v_{\max}\cdot {\mathrm{Joy}}_{y},\; & {\mathrm{Joy}}_{y} & =[-1,+1]\\ \omega_{d} & =\omega_{\max}\cdot {\mathrm{Joy}}_{x}, & {\mathrm{Joy}}_{x} & =[-1,+1] \end{array}$$

As a result, by minimizing the cost function, obstacle avoidance is achieved among other speed sets, ensuring safe driving, and the optimal linear and angular speed set is selected. These speed values are then sent to the motor driver via serial communication, providing the electric wheelchair’s safe movement according to the user’s request.

## 4. Experimental Result

**Figure 8:**
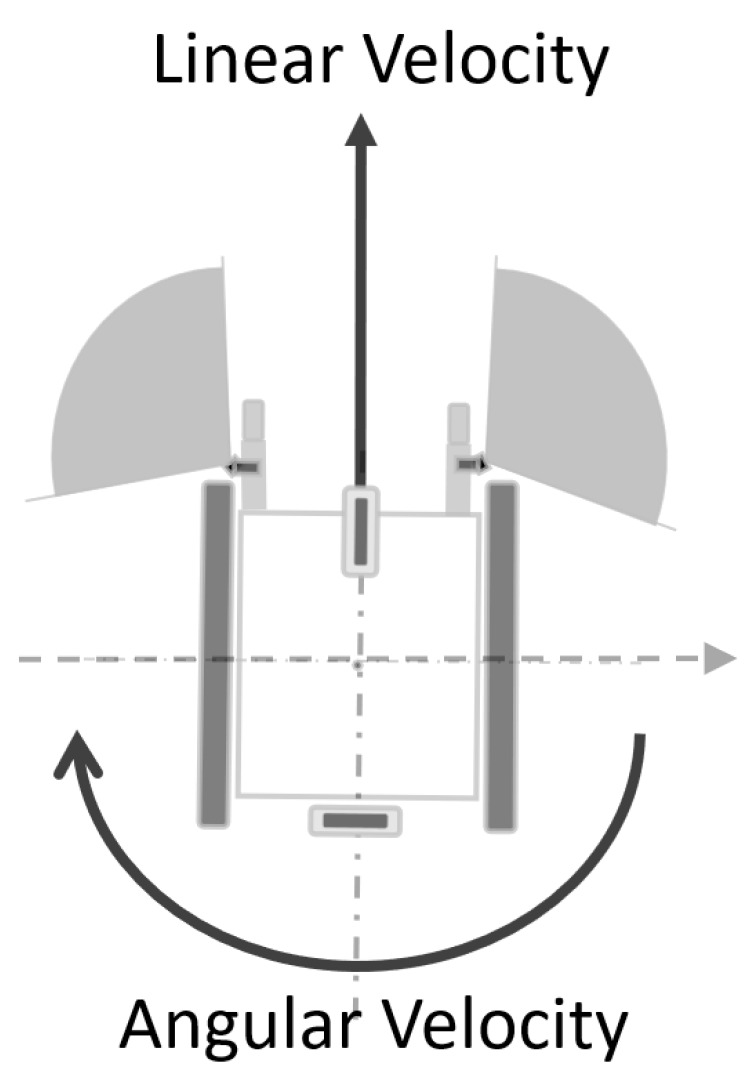
Angular velocity direction for the designed system.

For this study, three separate test scenarios have been prepared for the electric wheelchair expected to operate in real time under hazardous conditions and obstacles. Each of these three scenarios is tested individually and monitored with real-time graphics, and the test results are explained on the graph, while linear velocity is utilized in this study. Although the coordinate system of the wheelchair represents a known structure, the direction of angular velocity operates inversely. In other words, when the wheelchair moves to the left, the angular velocity value is positive, whereas when it moves to the right, the angular velocity value is negative. This situation is illustrated in Figure 8.

**Figure 9:**
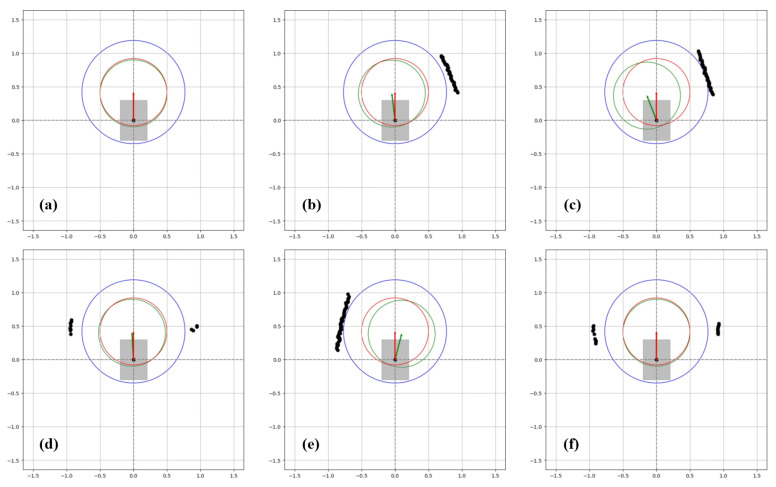
Detection of obstacles and avoidance during straightforward movement of the wheelchair.

**Figure 10:**
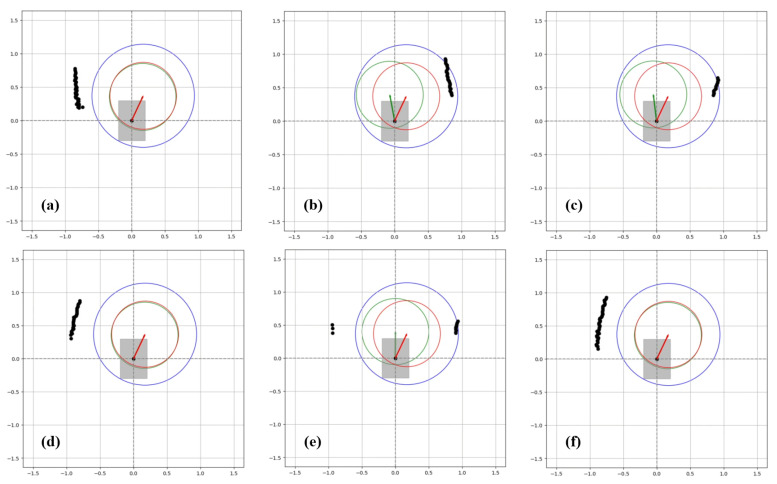
Detection of obstacles and avoidance while the wheelchair is moving obliquely.

**Figure 11:**
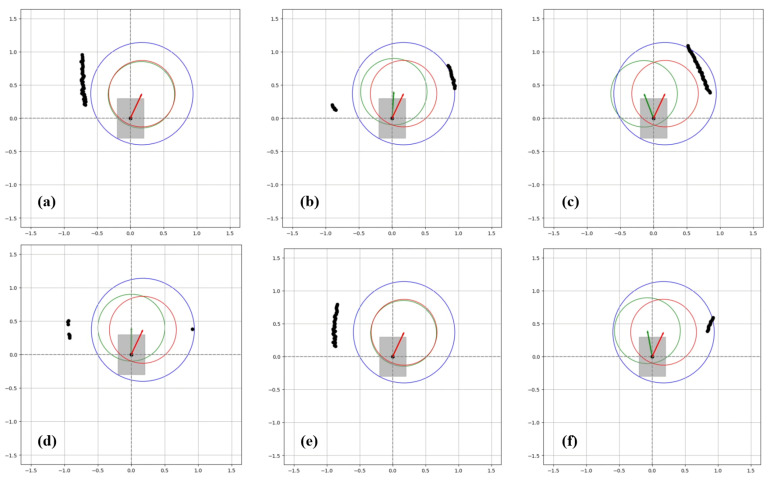
Detection of obstacles and avoidance while the wheelchair turns a corner.

One of the data obtained from the test results is the graphic system enabling real-time monitoring of the drive. These systems are described in Figure 9, Figure 10 and Figure 11, indicating that the algorithm exhibits real-time obstacle avoidance interaction with the distance. Additionally, each part denoted as a, b, c, d, e, and f in these figures indicates how the wheelchair is positioned and shows the outputs in different situations graphically. The red arrow signifies the desired velocity direction intended by the user via the joystick, and along with the red circle, it represents the predicted next position that the electric wheelchair needs to validate with the joystick data. The green arrow indicates the direction in which the system should move to avoid collision. That is, the direction of the arrow is expressed in terms of the linear and angular velocity of the wheelchair. Like the red arrow, this green arrow indicates the center of the green circle, representing the predicted next position of the electric wheelchair based on the algorithm’s output, and the length of the green arrow denotes the linear speed, while its direction signifies the angular speed. The blue circle represents a safe zone added longitudinally to ensure safe driving of the electric wheelchair. This safe zone aims to achieve safer driving.

**Figure 12:**
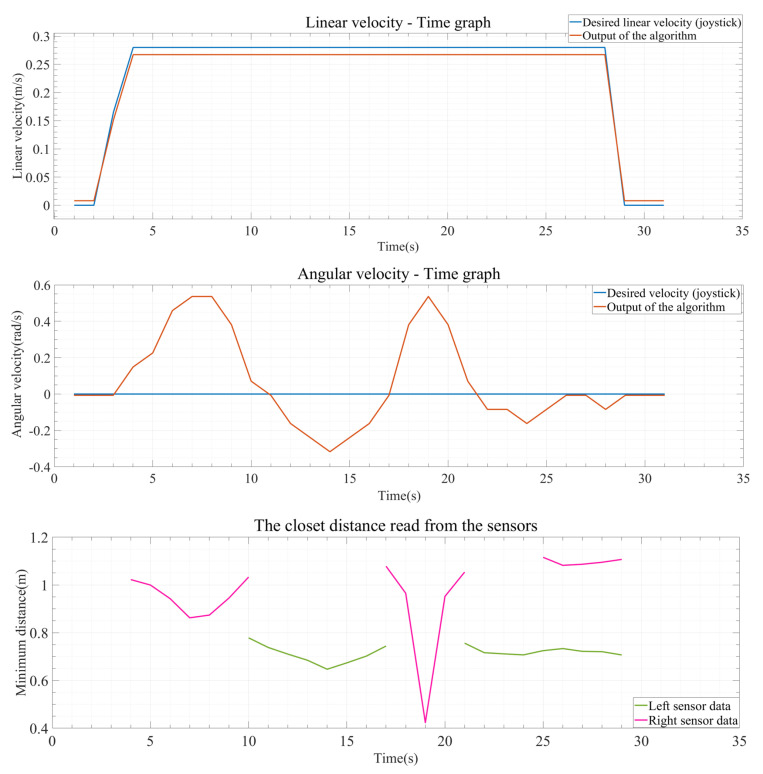
Output result graphs for experiment 1.

**Figure 13:**
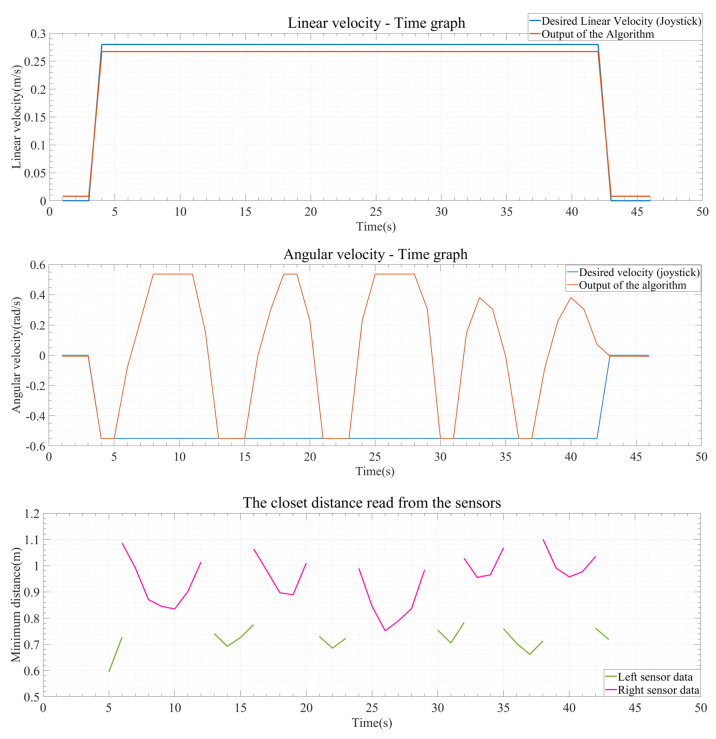
Output result graphs for experiment 2.

**Figure 14:**
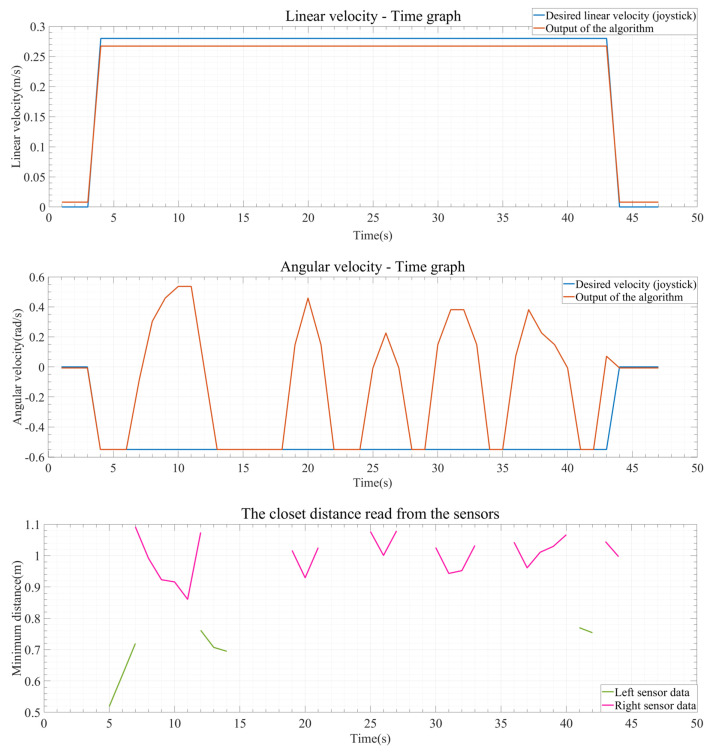
Output result graphs for experiment 3.

Another result obtained from these test results is the graphs generated from the collected data during the test. These graphs are shown in Figure 12, Figure 13 and Figure 14. The blue lines in the coordinate system represent the desired linear and angular velocity from the joystick. The orange lines represent the linear and angular velocity obtained from the sensor’s obstacle information as output from the algorithm. Additionally, in the distance graphs, the data from the right sensor (pink) and the left sensor (green) are separately shown on the coordinate system.

### 4.1. Experiment 1

**Figure 15:**
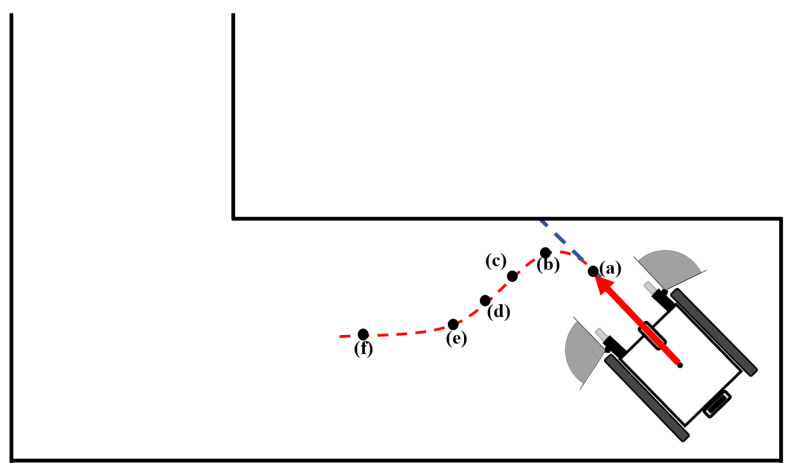
Experiment on collision avoidance when a wheelchair is positioned diagonally and moves towards a wall.

The first experiment, as depicted in Figure 15, demonstrates the electric wheelchair moving at maximum speed in a corridor, making a straight drive towards the wall using linear velocity.

Firstly, as seen in Figure 9a, since neither the right nor the left sensor detects any object, the electric wheelchair moves towards the next position with speed values equal to the desired joystick velocity and the output of the algorithm. When reaching point (b) and the right sensor detects the wall (obstacle), the next position indicated by the algorithm’s output directs the electric wheelchair towards the obstacle-free left side, contrary to the desired velocity. At point (c), as the right sensor continues to detect the wall, the algorithm’s output continues to move the electric wheelchair towards the left side. At point (d), since the electric wheelchair is in the middle of the two walls, both sensors detect the walls from a certain distance, but this distance does not cause a collision, so the wheelchair continues to move in the same direction as the data from the joystick. Due to the caster wheels located at the front of the wheelchair, sometimes, despite the straight command from the joystick, the wheelchair’s movement angle may deviate to the right or left. Figure 9e illustrates this situation, and when the left sensor detects the wall on the left side, the wheelchair tends to correct its movement again due to the developed algorithm. When no obstacle that would cause a collision is detected around the wheelchair, as seen again in point (f), it continues to move in the desired direction from the joystick. These graphs were recorded in real time during the wheelchair’s test drive.

In Figure 12, the state of the operating system after the test is completed is shown, with the data plotted on the graph. Since the joystick only moves straight forward, the linear velocity remains at its maximum level, and the linear velocity output from the algorithm also remains unchanged. One of the most important points here is the angular velocity and the minimum distance information read from the right and left sensors. In the distance graph, as the pink line representing the data from the right sensor approaches and begins to approach an object, the angular velocity value increases, indicating a movement away from the object. Furthermore, as the wheelchair moves away from the object, after a while, it starts to move towards the left wall as the wheelchair approaches the left wall, as indicated by the green line, and moves with small angular velocity values according to sensor data when the obstacle is avoided and is within the safe zone on both sides.

### 4.2. Experiment 2

**Figure 16:**
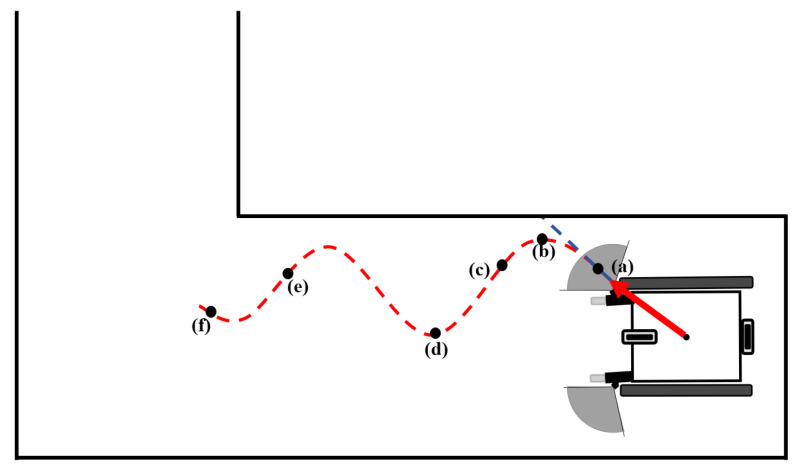
Experiment on preventing collision while rotating towards the wall during movement.

In the second experiment, as seen in Figure 16, the wheelchair is placed straight in the corridor, and the joystick commands a forward-right direction. In short, maximum values of linear and angular velocity were applied.

Firstly, as seen in Figure 10a, the wheelchair is orienting towards the desired direction indicated by the joystick. However, as the wheelchair approaches the right wall too closely, it enters the blue circle defined as the safe zone for objects, and through the algorithm, the wheelchair’s expected rightward movement with the joystick has been redirected to the left, ensuring avoidance from the obstacle as observed at points (b) and (c).

As seen in (d), when there are no obstacles on the right side, the algorithm moves the wheelchair with the speed values desired from the joystick, and as seen in (e), when it encounters the wall again, the angular velocity changes as a result of the algorithm for avoidance.

If we examine the outputs of this test, as seen in Figure 13, the linear velocity remains measured at its maximum level without any change. The most crucial aspect here is the changing angular velocity in response to obstacles. As observed from the graphs, initially, the wheelchair is moving towards the right direction, as indicated by the pink line, and detects the right wall (object), prompting it to move towards the left. Since these tests take place in a corridor, as it moves left, the left sensor starts working to detect the left wall, and when the minimum distance begins to decrease, the algorithm steers the wheelchair towards the right again by progressing with negative angular velocity, allowing it to avoid obstacles. As obstacles are perceived in this manner, the algorithm generates appropriate outputs to produce speeds suitable for the current environmental conditions, as depicted in the graphs of Figure 13.

### 4.3. Experiment 3

The third experiment aims to safeguard the user against potential collisions in situations where the user cannot adjust the turning angle or lacks awareness of what lies behind the turning point.

**Figure 17:**
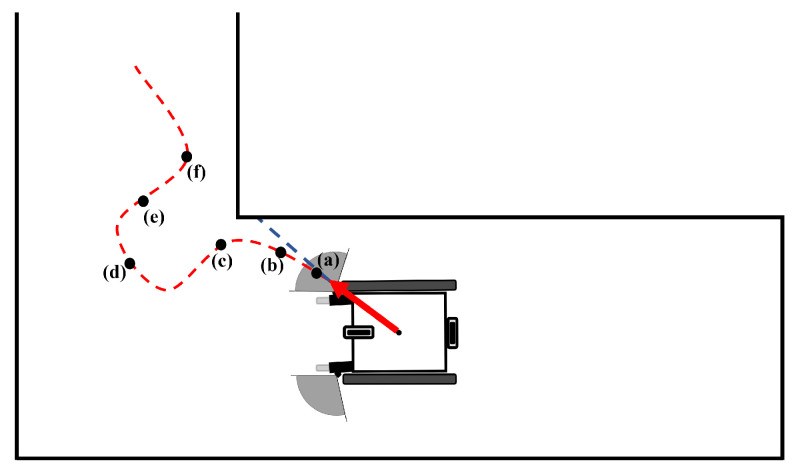
Experiment on collision avoidance while turning a corner.

In this experiment, similar to the second test, the wheelchair is placed in the middle of the corridor, but this time it is positioned to turn at a corner of the wall. The joystick is moved forward and to the right at maximum speed and illustrated in Figure 17.

As seen in Figure 11a, the wheelchair initially begins to move towards the desired position of the joystick, and shortly after the sensors detect the wall, a change in angular velocity results in the behavior of avoiding collisions. When the wall is closer, the angular velocity turns the wheelchair towards the left, as observed at points (b) and (c). At point (d), the point on the right represents the corner of the wall, and for a safe turn of the wheelchair, a lower angular velocity is used for collision avoidance. This is because it is expected to move appropriately according to the joystick command during avoidance.Once the corner is passed and no obstacles are detected on the right by the sensors, as seen in (e), the wheelchair moves in the desired position. In (f), the escape maneuver is repeated as the right sensor detects the wall again. The reason for this escape is due to the continuous indication of forward-right movement by the joystick throughout the test.

The outputs of this test are shown in Figure 14. Since there is no change in linear velocity, the output of the algorithm remains the same as the desired velocity. The crucial point here is the angular velocity. Examining the distance information, as the wheelchair turns towards the right, it demonstrates adaptive behavior by increasing angular velocity, along with the distance data from the left sensor indicated by the pink line. This behavior persists throughout the test, as clearly observed in the outputs.

## 5. Discussion

### 5.1. Discussion of Results

As observed from the graphs resulting from the experiments, the system tends to avoid objects in real time, thus preventing collisions. This system is designed for indoor use and demonstrates the operation of the wheelchair within indoor environments through three conducted experiments. These experiments can be perceived as more customized for extreme situations. Beyond these scenarios, the wheelchair operates like a regular wheelchair based on user joystick inputs, with the addition of an algorithm aimed at avoiding obstacles in indoor environments.

As depicted in the graphs, in obstacle situations, the algorithm appropriately adjusts the angular velocity to select the safest path based on sensor scans. This safe driving experience is evidenced by the graphs depicting angular velocity and the distance data from the right and left sensors.

### 5.2. Limitations and Future Works

In this study, tests were conducted in a specific area without changing environmental conditions to adapt the algorithm to the electric wheelchair and control the newly designed driving module in indoor spaces. This module and algorithm are designed for indoor environments where wheelchairs are frequently used. However, in these environments, there may sometimes be obstacles like stairs. Therefore, as a continuation of this study, an investigation could be carried out to detect points such as stairs when approaching without obstructing the user’s drive and ensuring that the electric wheelchair stops or makes a suitable movement to secure the drive. To incorporate such a feature into the system, further research is required. Additionally, testing the system on multiple wheelchairs could be considered to explore the weights of the values in the cost function.

In this paper, we conducted an experiment to evaluate the proposed algorithm’s utility. However, experiments in rapidly changing environments have not yet been carried out. We plan to refine the algorithm to ensure the wheelchair moves robustly across various environments. This will be the focus of our next research, which we will continue to pursue.

## 6. Conclusions

A removable driving module has been designed for an electric wheelchair, controlled by a joystick. The developed algorithm introduces real-time obstacle avoidance to the electric wheelchair. Considering the indoor usage of electric wheelchairs, there are limited studies that address both obstacle avoidance and unrestricted user movement. This study addresses the limitations of existing electric wheelchairs and obstacle avoidance approaches by developing a new algorithm, adapting the commonly used DWA for object avoidance in mobile robots. Through LiDAR sensors strategically placed in areas that do not restrict user movement, environmental information is provided. The algorithm operates based on user joystick commands, generating optimal speed values. The cost function, commonly used in traditional DWA, has been adapted for this study, considering speed and distance to objects. The algorithm selects speed sets that minimize the cost function, ensuring a safe drive. The system was then mounted on a real electric wheelchair, and experiments were conducted to analyze the algorithm’s performance.

Experimental results demonstrate significant improvements in maneuverability and user safety, highlighting the effectiveness of the adapted DWA algorithm in real-world applications. Additionally, the system’s modular design offers a flexible solution for enhancing mobility by facilitating easy integration with various wheelchair models. This research provides a practical and innovative approach to increasing the autonomy and safety of electric wheelchair users, making a substantial contribution to the field of assistive technology. Future studies will focus on enhancing the algorithm’s capabilities to handle more complex environments and dynamic obstacles. 

## Figures and Tables

**Table 1:** Specifications of the CygLiDAR sensor.

Detection Range	Resolution	Field of View	Measuring Speed
2D: 200 mm 8000 mm	2D: 1° (Angle)	2D/3D Horizontal: 120°	2D: 15Hz
3D: 50 mm 2000 mm	3D: 160 × 60 (Pixel)	3D Vertical: 65°	3D: 15Hz

## Data Availability

Data are contained within the article.

## Abbreviations

The following abbreviations are used in this manuscript:

DWA	Dynamic Window Approach
APF	Artificial Potential Fields
VFH	Vector Field Histogram
BCI	Brain–Computer Interface
MI	Motor Imagery
MSB	Most Significant Byte
LSB	Least Significant Byte
